# 7,200 years old constructions and mudbrick technology: The evidence from Tel Tsaf, Jordan Valley, Israel

**DOI:** 10.1371/journal.pone.0227288

**Published:** 2020-01-22

**Authors:** Danny Rosenberg, Serena Love, Emily Hubbard, Florian Klimscha

**Affiliations:** 1 Laboratory for Ground Stone Tools Research, Zinman Institute of Archaeology, University of Haifa, Haifa, Israel; 2 School of Social Science, University of Queensland, Australia; 3 The Archaeology Centre, University of Toronto, Canada; 4 Lower Saxony State Museum, Department of Research/Collections, Archaeology Division, Hanover, Germany; University at Buffalo - The State University of New York, UNITED STATES

## Abstract

The history of mudbrick production and construction in the southern Levant may be dated as far back to the Pre-Pottery Neolithic A. However, at many of the sites where mudbrick remains were noted, their preservation was poor, so investigation of their production and the related construction techniques in antiquity was precluded. The 7,200 year old (cal BP) site of Tel Tsaf, located in the Jordan Valley, is distinguished by outstanding preservation of mudbrick architecture, which enables us to delve into various issues related to mudbrick technology, construction and preservation. The present paper discusses some of the mudbrick features at Tel Tsaf and their characteristics and offers a comprehensive analytical study of the mudbricks from multiple contexts and phases. These demonstrate consistency in three of the four measured variables: magnetic susceptibility, organic content and calcium carbonate equivalent. The results of our study suggest that while we can identify morphometric variability between bricks and walls, by and large, a uniform composition characterized the tested assemblages without any temporal or spatial variability. This indicates that a single locally-sourced raw material was used and that recycling of old decayed mudbricks was likely practiced. The consistency of mudbrick-production during all phases of the occupation at Tel Tsaf and the absence of multiple recipes implies that a shared production and technological know-how was maintained for at least 500 years at the site.

## Introduction

Architecture is the largest and often best-preserved material cultural artefact to exhibit indicators of social organization, social differentiation and socio-technical continuity, and it may reflect social homogeneity following chronological and geographic boundaries. Architecture opens a social window into the prehistoric past, and the high preservation of mudbrick architecture in the Near East allows us to study this [1–5]. Architecture is also one of the best means to reconstruct ancient knowledge [6–10]. Recent studies have highlighted key aspects of ancient architecture, including the effect of architecture on social agencies and the agency of architecture itself [3–4]. Architecture can also convey power [5]. However, despite an abundance of theoretical approaches [6–10], quantifiable archaeological methods are often lacking, and prehistoric architecture is often neglected [11–12].

This is unfortunate as architecture actively communicates social values, norms and identity [13–15] especially in pre-literate societies [9–10, 16–20]. Further, while architecture presents physical evidence of past societies, architecture also exists beyond the physical environment, building on the foundations of actor-network concepts. Thus, we may study how people existed with buildings as well as in them [21] and how the related social norms were reproduced through time [6]. The interaction between humans and architecture is therefore a complex set of shifting dynamics to which object-based approaches may be applied [22].

Building materials and construction techniques can be indicators of wealth differentiation or act as other non-verbal modes of communication [7], a phenomenon detailed in multiple ethnographic and historic accounts [8, 23–24]. Architecture may also convey social status differentiation as well as variation in ideology [3, 5, 21]. The design and furnishing of architecture have been claimed to be similar to conspicuous consumption [25], and the way architecture is constructed certainly is a prime element of identity [26]. Architecture thus often serves to distinguish status, rank and power [5]. Nevertheless, other authors stress that the construction of architecture is often inclusive, masking such differences [27]. For instance, the design and organization of prehistoric architecture and construction may be based on both necessity and shared cultural traditions.

Due to the socially constructed nature of spaces and the ability of architecture to structure space [28], different architectural concepts allow the study of prehistoric identity detached from mobile elements of material culture [26]. Individual and collective identities are expressed and contested through the non-verbal communication of architecture; archaeological methods may then quantify this, recovering social meaning from the fabric of the house [8, 16, 26, 29–31]. For example, prehistoric architecture is frequently used as a medium to demonstrate wealth and power as a manifestation of authority and command over human and material resources [32–33]. The construction materials may also be analyzed as they too connect people to places through the houses they build. Construction material may also elucidate on a variety of related aspects including craft specialization, management of resources and labor organization. In particular, these are visible in mudbricks through the analysis of textures, tempering agents and raw materials (e.g. [34–36]).

Furthermore, the analysis of the *chaîne opératoire* allows one to model logistics and division of labor within a community [4, 37–38]. This information adds an additional layer of meaning into understanding prehistoric architecture by informing on the physical environment and how it was exploited through the social choices displayed by the architects and the producers of construction material [39].

Mudbrick architecture is imbued with cultural information that reflects human choices (e.g. [35, 40–44]). Each choice possesses information about collective and individual cultural practices [37]. Therefore, studies on mudbrick production may allow the identification of shared recipes just as studies on ceramic technology can discern between two potters sharing a single source material [45–46]. However, mudbricks are unfortunately one of the least studied architecture features in the prehistoric southern Levant. This is mainly due to the lack of well-preserved mudbrick remains at most prehistoric sites in the region. There is minimal research concerned with mudbrick construction and technology, and with few exceptions [47], mudbrick architecture is only identified as a superstructure paired with a better preserved stone foundation.

However, sun-dried mudbricks (adobe) formed from a mixture of sand, clay, water and frequently temper (e.g. chopped straw and chaff branches), was the most common method for constructing earthen buildings for millennia. These mudbrick constructions rely on readily available materials, are relatively quick and easy to make and maintain relatively moderate inside temperature (e.g. [41, 48]). Nevertheless, they required frequent maintenance as they are vulnerable to decay, mainly due to rain [41, 48].

Mudbricks seem to appear for the first time in the southern Levant as early as the Pre-Pottery Neolithic A period (PPNA) (e.g. [49–54]), and they continued to be used in the region until recently (see [55]). Mudbrick constructions and mudbricks as architectural features reflect several specific choices entailed in production, construction and maintenance. The study of these choices demands a site with a high degree of preservation. One of the best candidates to explore these issues is the Middle Chalcolithic settlement of Tel Tsaf, located in the Jordan Valley, Israel. At the site, architectural features and installations were constructed from sun-dried mudbricks that are well preserved due to the climatic conditions. The present paper discusses mudbrick production, mudbrick-related construction techniques and various architectural features at Tel Tsaf. We will demonstrate that shared technologies were used by the inhabitants of the site for centuries and that the geological settings of the area dictated construction techniques and practices at the site.

## Tel Tsaf

**Fig 1:**
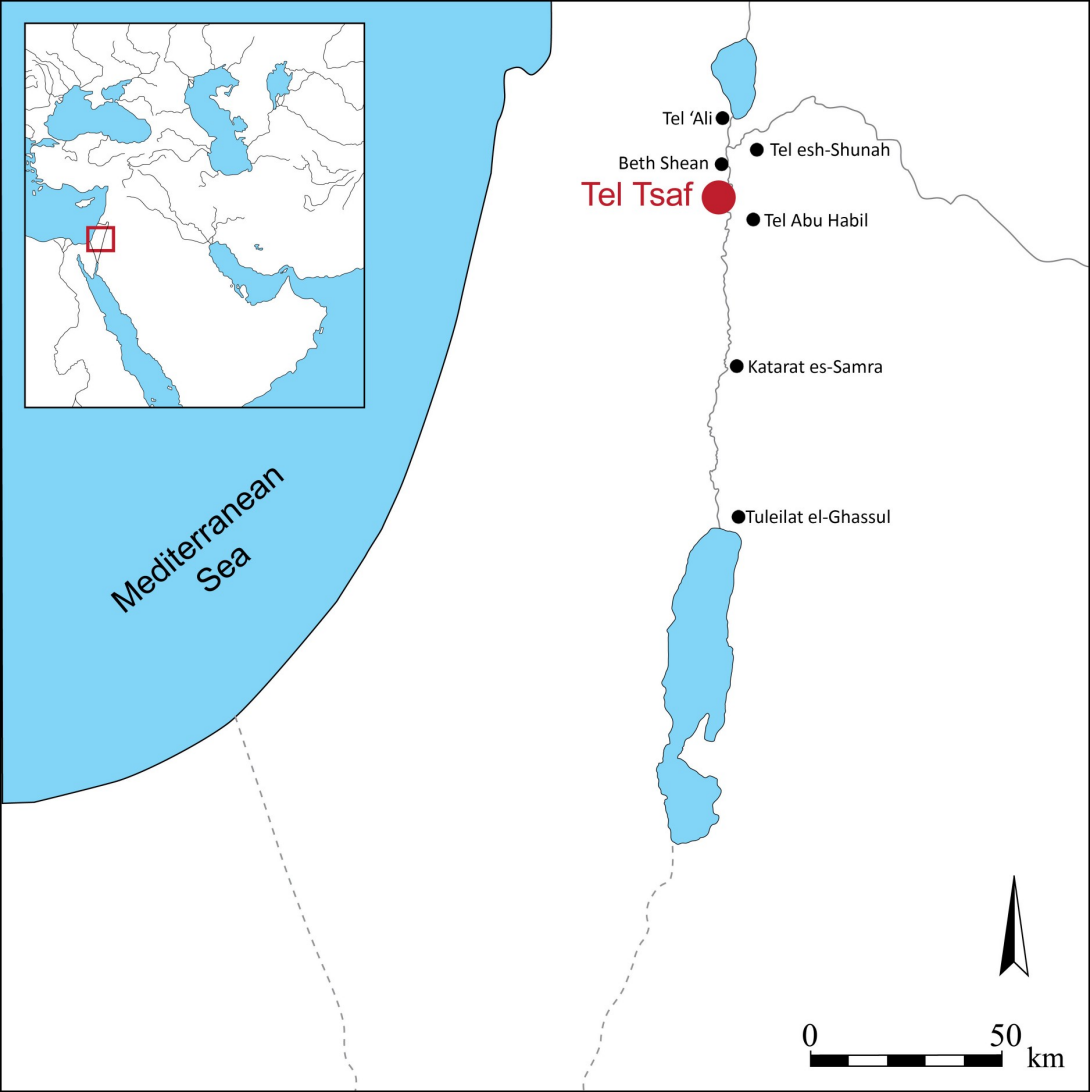
The location of Tel Tsaf and other 6^th^ and 5^th^ Millennia cal BCE sites.

Tel Tsaf is located in the middle Jordan Valley (Fig 1) and is dated between the Early Chalcolithic and Late Chalcolithic periods, ca. 5200–4700 cal BCE, also referred to as the Middle Chalcolithic [56–60]. The site consists of three hills that were formed by laminate layers of sediment deposited by the Pleistocene Lisan lake [61–63]. The predominant deposits are dolomite, calcite and clay from the shoulders of the lake basin in addition to windblown quartz and calcite [64]. The modern surface sediment is composed of a mixture of aeolian and fluvial deposits, with alluvial material derived from local limestone and calcitic sand, silt and clay. These Lisan sediments and alluvial deposits run parallel to the Jordan River through much of the Jordan Valley, and the surface geology has remained relatively stable during the mid to late quaternary.

**Fig 2:**
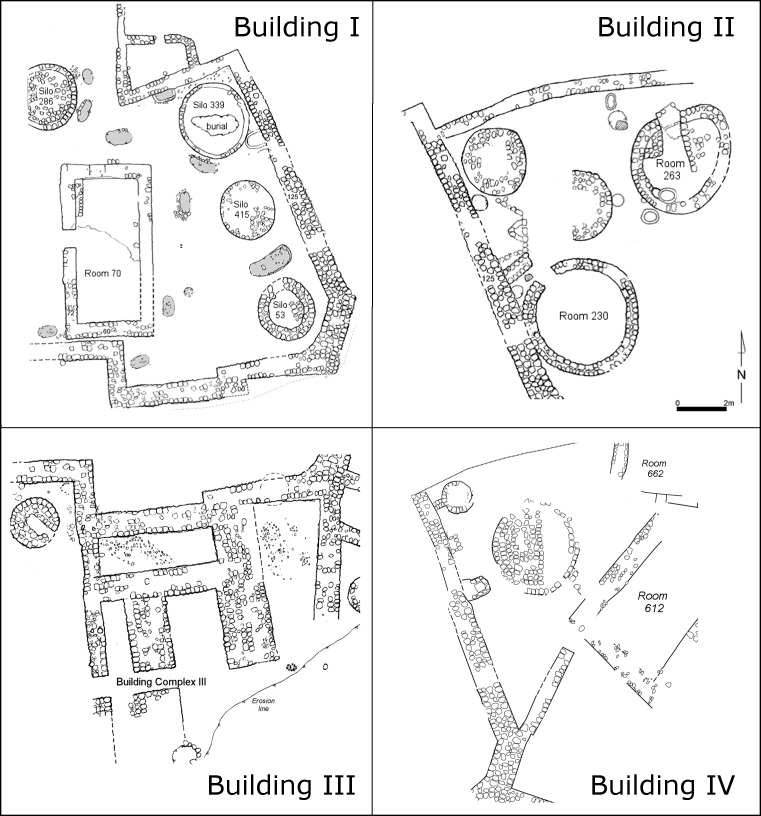
Major architecture units in Tel Tsaf, Area C (after [58, 70]).

The site was first reported in the 1950’s [65]. Small-scale excavations (ca. 100 m^2^) were conducted from 1978–1980 [66], revealing Pottery Neolithic and Early Chalcolithic strata. Later excavations from 2004 to 2007 exposed approximately 800 m^2^ [58, 60, 67–72]. In the main excavation area (Area C), remains of four large courtyard buildings were found (Buildings I-IV); these are characterised by rooms enclosed by courtyards that contain various installations (Fig 2), identified primarily as silos and cooking facilities. Burials were discovered in and around the silos [58], one of which, in Building CI, contained a young woman with 1668 beads arranged in a chain around her waist and a small copper awl [73]. Other finds included many objects indicative of long-distance trade, such as obsidian, Ubaid pottery, beads, shells and minerals [58, 67, 70, 74].

The current project was initiated in 2013 [59, 75]. The project analyzes the temporal and spatial attributes of social and economic variations at the site, documents dietary changes, explores the early lower strata of the settlement, and conducts a paleoenvironmental and ecological study to shed new light on how the Jordan Valley resources were utilized at the site. This in turn reflects on the Neolithic-Chalcolithic transition in the Jordan Valley and the contextual, social and ecological settings involved in the establishment of the Mediterranean diet in the region. Central to the new project is the interrelation between the complex architecture and building technology with the rich material culture assemblages and the environmental settings. By studying the technology of a large sample of single mudbricks, technical choices as well as constraints put upon the producers by a variety of factors. In a further step, the results are discussed against the background of the architecture of Tel Tsaf.

## Materials and methods

**Fig 3:**
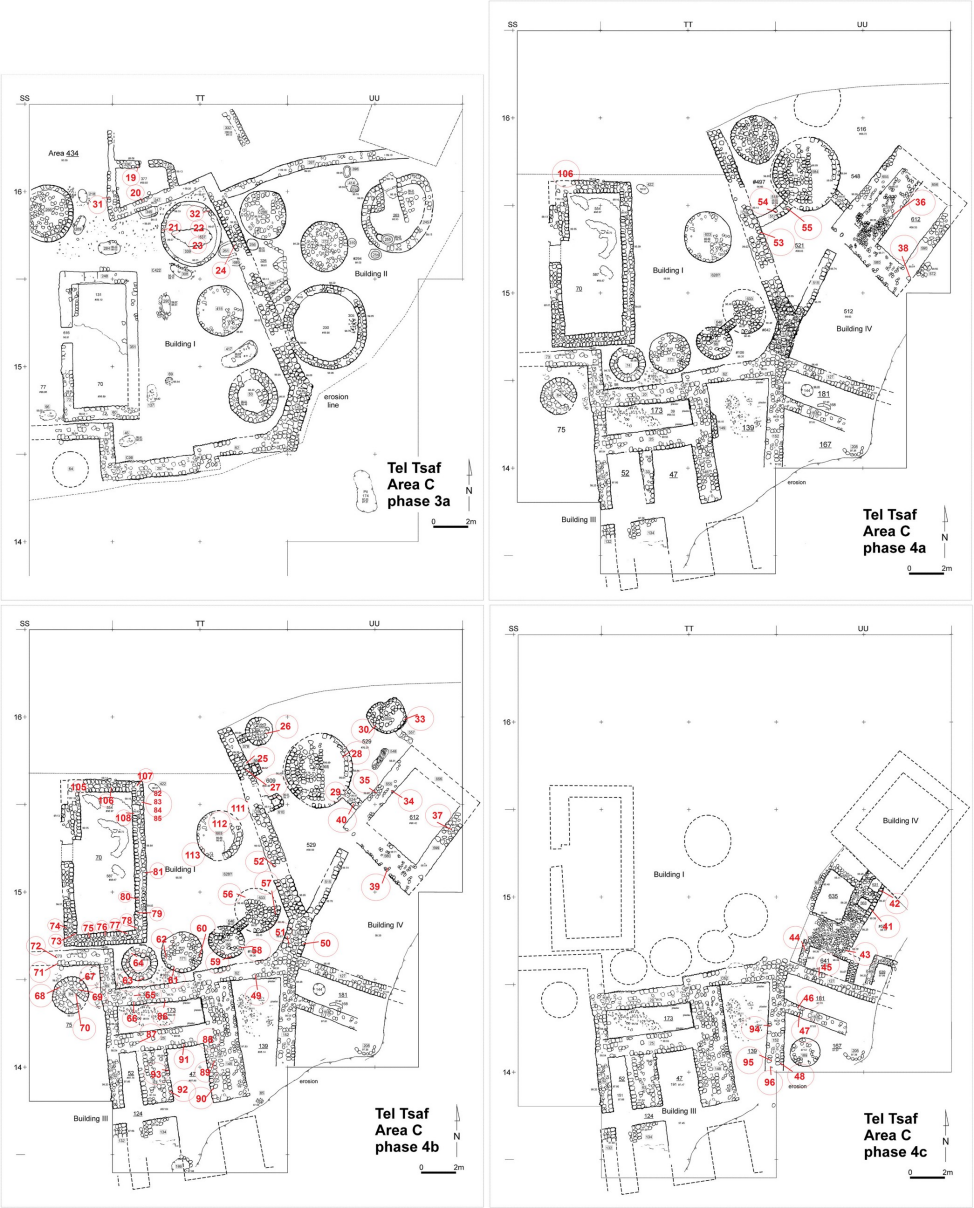
Mudbrick sampling locations in Area C.

Work on various architectural features is on-going and here we describe only the main characteristics of the construction techniques. To gain a wider understanding of the culturally-modified sediments used in construction at Tel Tsaf, unconsolidated brick samples were collected (all sampled were collected during the 2014 season at Tel Tsaf, under the IAA license G-8/2014) from all available architecture units and installations (Table 1), including the four buildings, partition walls, courtyard walls and silos (Fig 3). 25 mudbricks underwent petrographic analysis at the University of Toronto (Table 2), petrographic analysis was also carried out on mudbrick fragments visible in micromorphological samples collected during the 2006/2007 seasons, [71, 76], and 111 samples were collected and sent to the Geoarchaeology Laboratory at University of Queensland for further analysis. The analysis included magnetic susceptibility, loss on ignition, particle size analysis by hydrometer and RGB digital color [35]. These methods were selected based on prior success where texture, organic loss, calcium carbonate equivalent content and the mass specific magnetic susceptibility value were demonstrated as meaningful discriminating variables [29, 77–78]. Exploitation of locally-sourced sediments for mudbrick production does not always result in significant differences in mineralogy or geochemical signatures [77, 79]; therefore, high-resolution analytical techniques to investigate mineralogy and geochemistry were not employed.

**Table 1:** The mud bricks sampled for geoarchaeological analysis.

Sample#	Area	Phase	Building	Context	Wall
**Tsaf1**	C	4a-4b	I	Room70	W705A
**Tsaf2**	C	4a-4b	I	Room70	W705B
**Tsaf3**	C	4a-4b	I	Room70	W711A
**Tsaf4**	C	4a-4b	I	Room70	W711B
**Tsaf5**	C	4a-4b	I	Partition wall, south of Room 70	W707A
**Tsaf6**	C	4a-4b	I	Partition wall, south of Room 70	W707B
**Tsaf7**	C	?	I	L763	L784
**Tsaf8**	C	?	I	L763	L784
**Tsaf9**	C	?	I	L763	L784
**Tsaf10**	C	?	I	L763	L784
**Tsaf11**	C	?	I	L763	L783
**Tsaf12**	C	?	I	L763	L783
**Tsaf13**	C	?	I	L763	L783
**Tsaf14**	C	?	I	L763	L783
**Tsaf15**	C	?	I	CANCELLED	CANCELLED
**Tsaf16**	C	?	I	L763	L782
**Tsaf17**	C	?	I	L763	L782
**Tsaf18**	C	?	I	L763	L782
**Tsaf19**	C	3a	I	Room 377	L326
**Tsaf20**	C	3a	I	Room 377	L247
**Tsaf21**	C	3a	I	Silo 339	Outer wall
**Tsaf22**	C	3a	I	Silo 339	
**Tsaf23**	C	3a	I	Silo 339	
**Tsaf24**	C	3/4	I/IV	Partition wall	W125
**Tsaf25**	C	3/4	I/IV	Partition wall	W125
**Tsaf26**	C	4b	IV	Silo 565	
**Tsaf27**	C	4b	IV	L566	
**Tsaf28**	C	4a/4b	IV	Silo 568	
**Tsaf29**	C	4a/4b	IV	Silo 568	
**Tsaf30**	C	4b	IV	Silo 550	
**Tsaf31**	C	3a	I	Room 377	Western wall
**Tsaf32**	C	3a	I	Silo 339	Outer wall
**Tsaf33**	C	4b	IV	Silo 550	
**Tsaf34**	C	4b	IV	Room 612	W655
**Tsaf35**	C	4b	IV	Room 612	W655
**Tsaf36**	C	4a/4b	IV	Room 612	533
**Tsaf37**	C	4b	IV	Room 612	W599
**Tsaf38**	C	4a/4b	IV	Room 612	W590
**Tsaf39**	C	4b	IV	Room 612	W580
**Tsaf40**	C	4a-4b	IV	?	W524
**Tsaf41**	C	4c	IV	L563	Eastern wall
**Tsaf42**	C	4c	IV	L531	Eastern wall
**Tsaf43**	C	4c	IV	L632	
**Tsaf44**	C	4c	IV	Room 641	Western wall
**Tsaf45**	C	4	IV	?	W127
**Tsaf46**	C	4	IV	Room 181	W163
**Tsaf47**	C	4	IV	Room 181	W163
**Tsaf48**	C	4	IV	?	W152
**Tsaf49**	C	3/4	I	?	W62
**Tsaf50**	C	3/4	I/IV	Partition wall	W143
**Tsaf51**	C	3/4	I/IV	Partition wall	W143
**Tsaf52**	C	3/4	I/IV	Partition wall	W125
**Tsaf53**	C	3/4	I/IV	Partition wall	W125
**Tsaf54**	C	4a	IV	?	W517
**Tsaf55**	C	4a	IV	?	W517
**Tsaf56**	C	4	I	Silo 633	
**Tsaf57**	C	4	I	Silo 633	
**Tsaf58**	C	4	I	Silo 66	
**Tsaf59**	C	4	I	Silo 66	
**Tsaf60**	C	4	I	Silo 177	
**Tsaf61**	C	4	I	Silo 177	
**Tsaf62**	C	4	I	Silo 74	
**Tsaf63**	C	4	I	Silo 74	
**Tsaf64**	C	4	I	Silo 74	
**Tsaf65**	C	4	I/III	Partition wall	W20
**Tsaf66**	C	4	I/III	Partition wall	W20
**Tsaf67**	C	4	III	Silo 64	
**Tsaf68**	C	4	III	Silo 64	
**Tsaf69**	C	4	III	Silo 64	
**Tsaf70**	C	4	III	Silo 64	
**Tsaf71**	C	3/4	I/III	Partition wall	W73
**Tsaf72**	C	3/4	I/III	Partition wall	W73
**Tsaf73**	C	3/4	I	Room70	W72
**Tsaf74**	C	3/4	I	Room70	W72
**Tsaf75**	C	3/4	I	Room70	W60
**Tsaf76**	C	3/4	I	Room70	W60
**Tsaf77**	C	3/4	I	Room70	W60
**Tsaf78**	C	3/4	I	Room70	
**Tsaf79**	C	3/4	I	Room70	W105
**Tsaf80**	C	3/4	I	Room70	W105
**Tsaf81**	C	3/4	I	Room70	W105
**Tsaf82**	C	3/4	I	Room70	W105
**Tsaf83**	C	3/4	I	Room70	W105
**Tsaf84**	C	3/4	I	Room70	W105
**Tsaf85**	C	3/4	I	Room70	W105
**Tsaf86**	C	4	I/III	Partition wall	W20
**Tsaf87**	C	4	III	?	W25
**Tsaf88**	C	4	III	?	W25
**Tsaf89**	C	4b-4c	III	Room 47	W148
**Tsaf90**	C	4b-4c	III	Room 47	W148
**Tsaf91**	C	4	III	?	W25
**Tsaf92**	C	4b-4c	III	?	W63
**Tsaf93**	C	4b-4c	III	?	W63
**Tsaf94**	C	4	III	?	W152
**Tsaf95**	C	4	IV	?	W152
**Tsaf96**	C	4	IV	?	W152
**Tsaf97**	C	4b-4c	III	?	W63
**Tsaf98**	C	4b-4c	III	?	W63
**Tsaf99**	C	?	?	?	
**Tsaf100**	C	?	?	?	W73
**Tsaf101**	C	?	?	?	W73
**Tsaf102**	C	?	?	?	W788
**Tsaf103**	C	?	?	L809	
**Tsaf104**	C	?	?	L809	
**Tsaf105**	C	3/4	I	Room70	Northern wall
**Tsaf106**	C	3/4	I	Room70	Northern wall
**Tsaf107**	C	3/4	I	Room70	Northern wall
**Tsaf108**	C	3/4	I	Room70	
**Tsaf109**	C	?	?	?	
**Tsaf110**	C	3/4	I	Room70	Northern wall
**Tsaf111**	C	4	I	Silo 603	

**Table 2:** The mud bricks sampled for petrographic analysis.

Sample#	Area	Phase	Building	Context
**TS06-2**	C	3	I	Room 70 interior
**TS06-3**	C	3	I	Room 70 interior
**TS06-3**	C	3	I	Room 70 interior
**TS06-9**	C	3	I	Courtyard
**TS06-10**	C	3	I	Courtyard
**TS06-13**	C	3	II	Room 230 interior
**TS06-14**	C	3	II	Room 230 interior
**TS06-15**	C	3	II	Room 230 interior
**TS07-2**	C	3	II	Room 263 interior
**TS07-20**	C	4	I	Room 70 interior
**TS07-33**	C	3	I	Room 70 SE wall
**TS07-34**	C	3	I	Silo 339 interior
**TS07-36**	C	3	I	Silo 339 interior
**TS07-37**	C	3	I	Silo 339 interior
**TS07-40**	C	4	II	Room 612 interior
**TS07-41**	C	4	II	Room 612 interior
**TS07-42**	C	4	II	Room 612 interior
**TS07-43**	C	4	II	Room 612 interior
**TS07-44**	C	3	II	Silo 271 section
**TS07-45**	C	3	II	Silo 271 section
**TS07-46**	C	4	II	Silo 271 section
**TS07-47**	C	4	II	Silo 271 section
**TS07-48**	C	4	II	Silo 271 section
**TS07-49**	C	4	II	Silo 271 section
**TS07-50**	C	4	II	Silo 271 section

A Bartington MS3 environmental magnetic susceptibility meter was used to relatively quantify the iron (Fe) content of sediments. Multiple high and low-resolution measurements were averaged to obtain mass-specific magnetic susceptibility values at a 1.0-k interval scale [80]. Relative amounts of organic and calcium carbonate present were quantified using loss on ignition [81–82]. Samples of 10 g of sediment were ignited at sequential temperatures of 105˚C, 550˚C and 900˚C. Particle size was determined with a hydrometer method at 152-H calibration. 30 grams of each sample was oven dried at 105˚C to remove water and soaked in a sodium hexametaphosphate solution for 24 hours. These were tested at standard intervals (following [83]) in a constant 20˚C laboratory. The remaining sand fraction was captured in a 64 μm screen, washed to remove silts and clay and air dried. Macroscopic analysis at 5x magnification was conducted on sieved 250 μm and 125 μm sand fractions. Mudbrick data was analyzed, and multivariate analysis, specifically principle component analysis, was conducted using JMP 13 statistical software.

## Results

### The main architectural features at Tel Tsaf

Four courtyard buildings were excavated [58, 67, 69–70, 74, 84]. While the full extent of these structures was not completely exposed, variations between architectural features were noted. These structures were made of sun-dried mudbrick walls and packed earth floors; stones were rarely used in construction.

**Fig 4:**
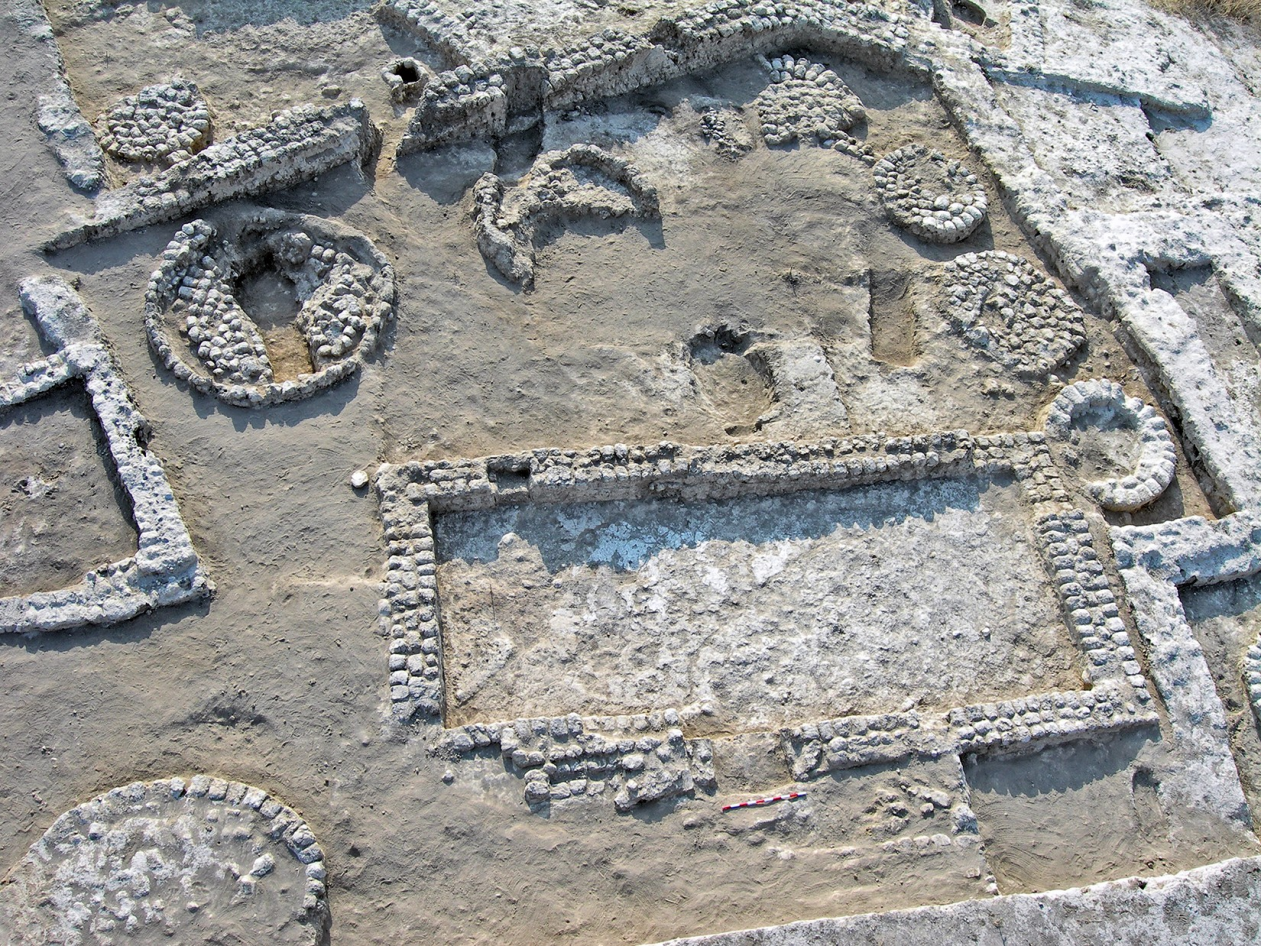
Building CI in Tel Tsaf Area C, a view to southeast (courtesy of Y. Garfinkel).

Building CI (Phases 3 and 4, Fig 4) consists of a large enclosure and an open courtyard bordered by walls in the south and east; the north and west are currently beyond the excavated area [74]. Excavation has revealed two architectural phases within the complex (Phases 3 and 4 of the overall site sequence; see [58, 70]). A rectangular room (Room C70) was revealed inside the enclosure with its entrance in the west. Room C70 (interior area ca. 27 m^2^) is surrounded by round structures, most of which were probably used as silos [58, 70], as well as cooking pits and ovens scattered over the courtyard. The entrance to the room was probably via the main courtyard [75, 84]. Apart from a slight change in the orientation of Room C70 and the location and number of silos, the general plan of the complex did not change between the two phases. During the renewed excavations, several more phases were discerned, suggesting that Building CI was preceded by earlier Middle Chalcolithic occupation levels.

Building CII (Phase 3) is located east of Building CI and consists of a single architectural phase divided into two sub-phases. The courtyard wall defines the northern and western extremes of the complex, but erosion has greatly affected the eastern and southern portions. Two round rooms were noted in its upper phase, with their opening facing north. Three round rooms used as residential units were noted in its lower phase [58]. The lower parts of the round rooms were sunken below the courtyard floor level. Two round silos were noted as well as another large structure (Locus C568) that was also interpreted as a silo. Its size (ca. 4 m in diameter) and archaeological characteristics might however also suggest other functions. Two of the burials found so far at Tel Tsaf are associated with this structure; burial C518 was inserted in the floor of the earlier phase, and a jar burial (Burial C310) was deposited next to its eastern side [58].

Building CIII (Phase 4) lies in the southern part of Area C directly south of Building CI and is poorly preserved. However, several architectural units were noted [58, 74]. The basic plan of the building includes a western open courtyard, two rectangular rooms in the north and east and a series of narrow chambers flanking a ‘corridor’ in the south. Some of the walls have a width of up to 1.8 m.

Building CIV (Phase 4) is located beneath Building CII and is characterized by similar courtyard walls but with a different, poorly defined interior arrangement [58, 74]. It consists of a rectangular room (5 m × 5 m), as well as three round silos and cooking installations. As under Building CI, test trenches revealed earlier Middle Chalcolithic occupation levels.

### Construction techniques at Tel Tsaf

**Fig 5:**
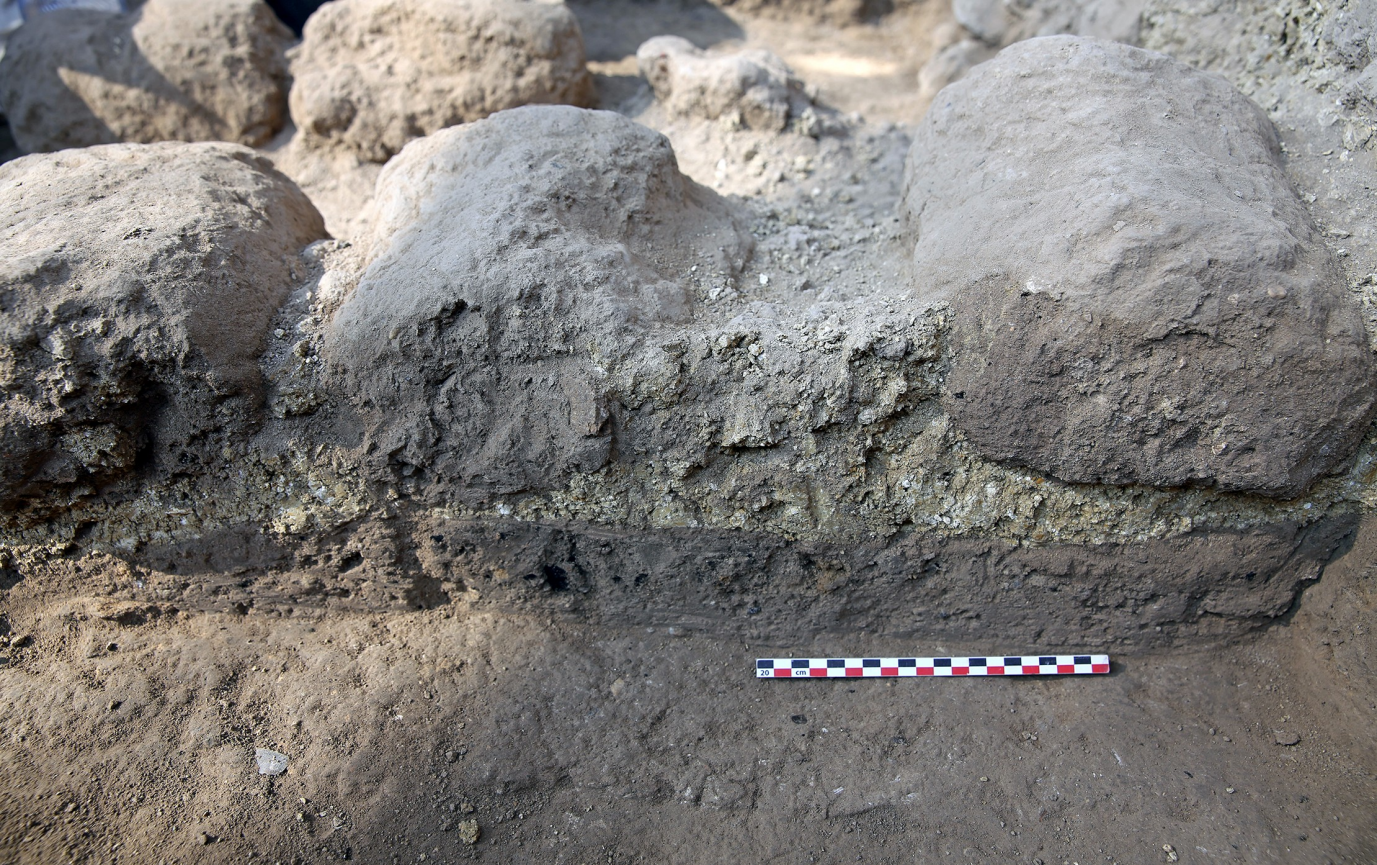
A wall and its cementing material.

Most architectural features at Tel Tsaf are made of sun-dried mudbricks that were cemented by mud-plaster, lime-plaster or other types of mortar (Fig 5). While this is due to plentiful availability of these raw materials, it also reflects scarce utilization of other materials that were easily available, specifically stones. Stones, such as limestone or basalt, were rarely used as building material, included in walls or used as stone foundations. Wood, reeds and other flora remains were found at Tel Tsaf as well (e.g. [59, 85]), and some of these may have originated as construction material used in roofing and superstructures.

If more recent mudbrick houses (e.g. [51, 86–87]) are used as an analogy, the houses at Tel Tsaf would have had flat roofs supported by horizontally placed wooden beams; they were less likely supported by wood logs placed in postholes. As most of the features (e.g., walls, silos) comprising the interiors of Buildings I-IV at Tel Tsaf are not set far from each other, frequently only 100–200 cm apart, extremely long wooden beams or logs were not a necessity. In some cases, mud fragments with linear imprints of reeds or wood, may represent such roofing as are chunks of phytoliths noted in some cases. Possible postholes were noted; however, these are not always clearly identified as such.

**Fig 6:**
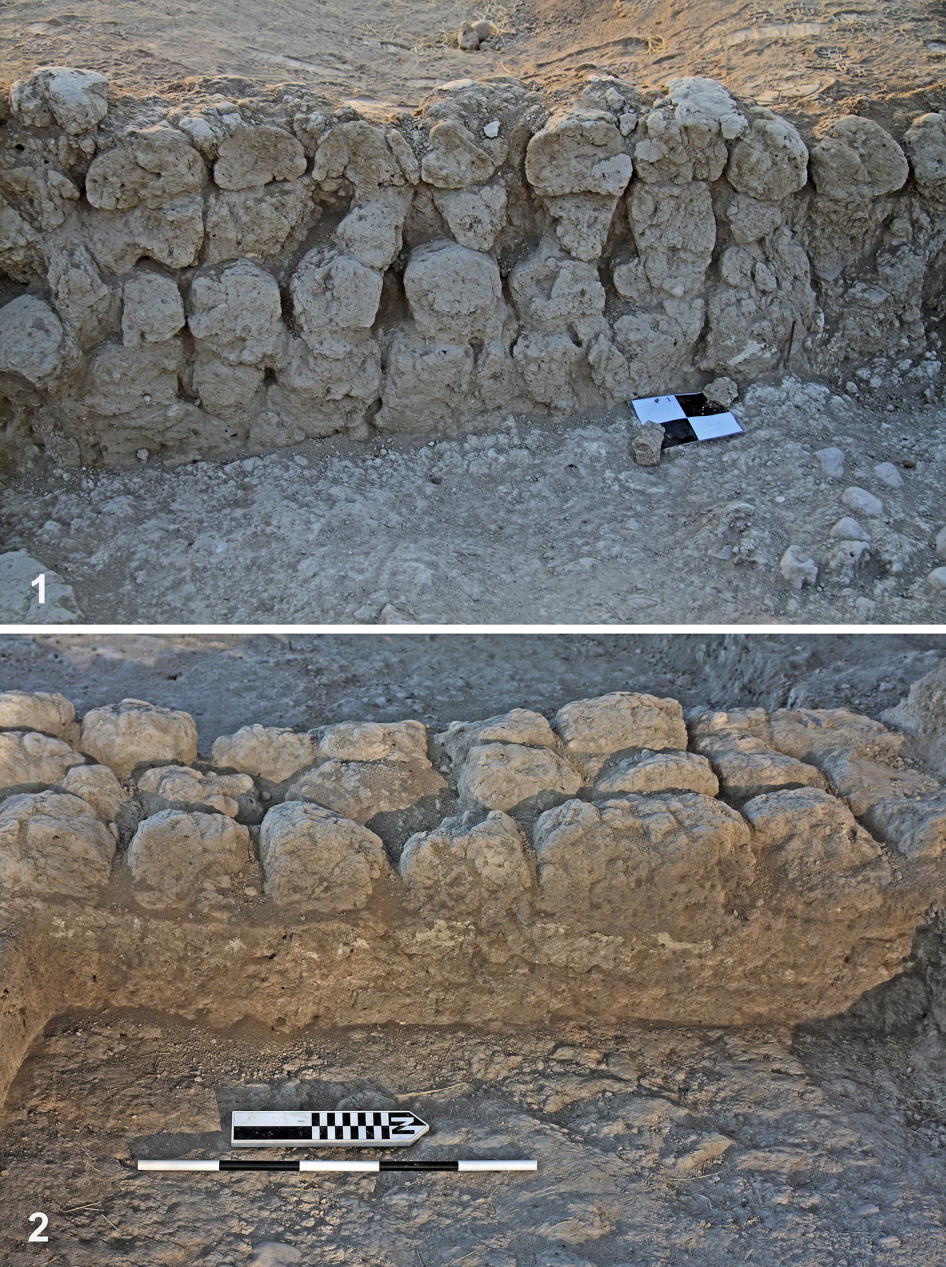
Mudbrick walls (Room C70).

The walls [84] are straight or rounded. The width of the straight walls generally ranges between 60 and 100 cm (and rarely more than that), and these were constructed from three rows of mudbricks (e.g., Room C70). The two outer rows were built with the narrow side of the bricks facing the interior and exterior of the construction (“headers”), and the inner row was laid perpendicular to the outer rows (“stretchers”) (Fig 6). Thicker walls were constructed from several rows in various arrangements of headers and stretchers, while thinner walls were built of two rows of bricks laid as stretchers or as headers [84]. Single-row mudbrick walls were used mainly for installations.

Different arrangements of mudbricks were used for specific construction types. Rounded walls were used for silos and rooms of unclear function. This can best be seen in Building CII that was built of two parallel rows of mudbricks laid as headers [84]. Silos were circular and constructed of mudbricks, usually with a massive circular foundation made of bricks in the center and a wall that was only a single brick thick (based on the few preserved cases usually to a height of one course). Rarely were these dug into the ground. In some cases, the use of bricks standing on their narrow side was noted.

**Fig 7:**
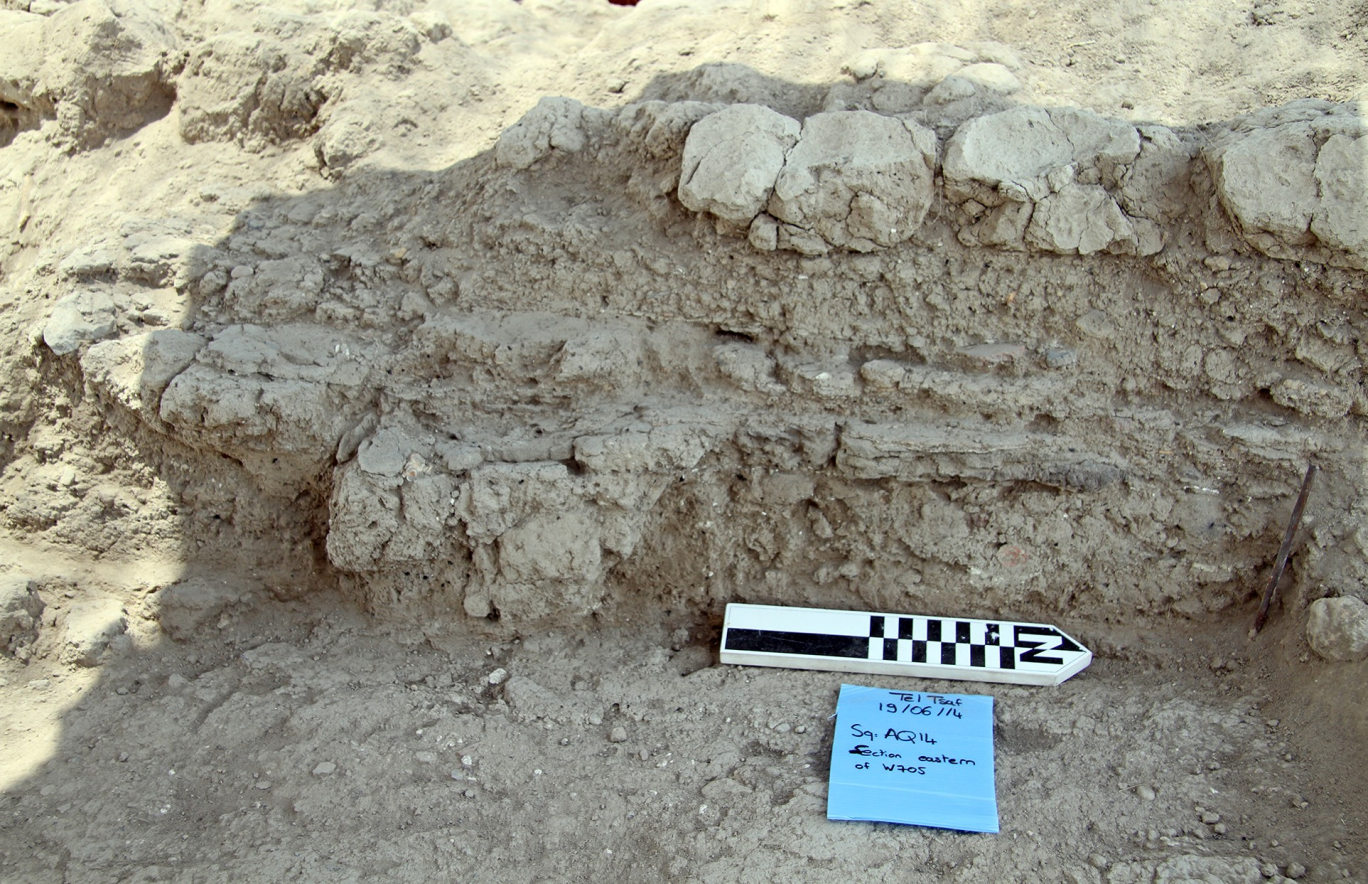
Plastered floor levels in the section of Room C70.

Floors were constructed in various methods using different materials for their composition and leveling. Their foundations were made of angular cobbles, river stones or small pebbles. Pebbles and cobbles were used as pavement, usually for limited areas. Beaten earth floors were used for inside and outside areas alike. Repeated plastering of floors was also noted (Fig 7), suggesting regular maintenance.

**Fig 8:**
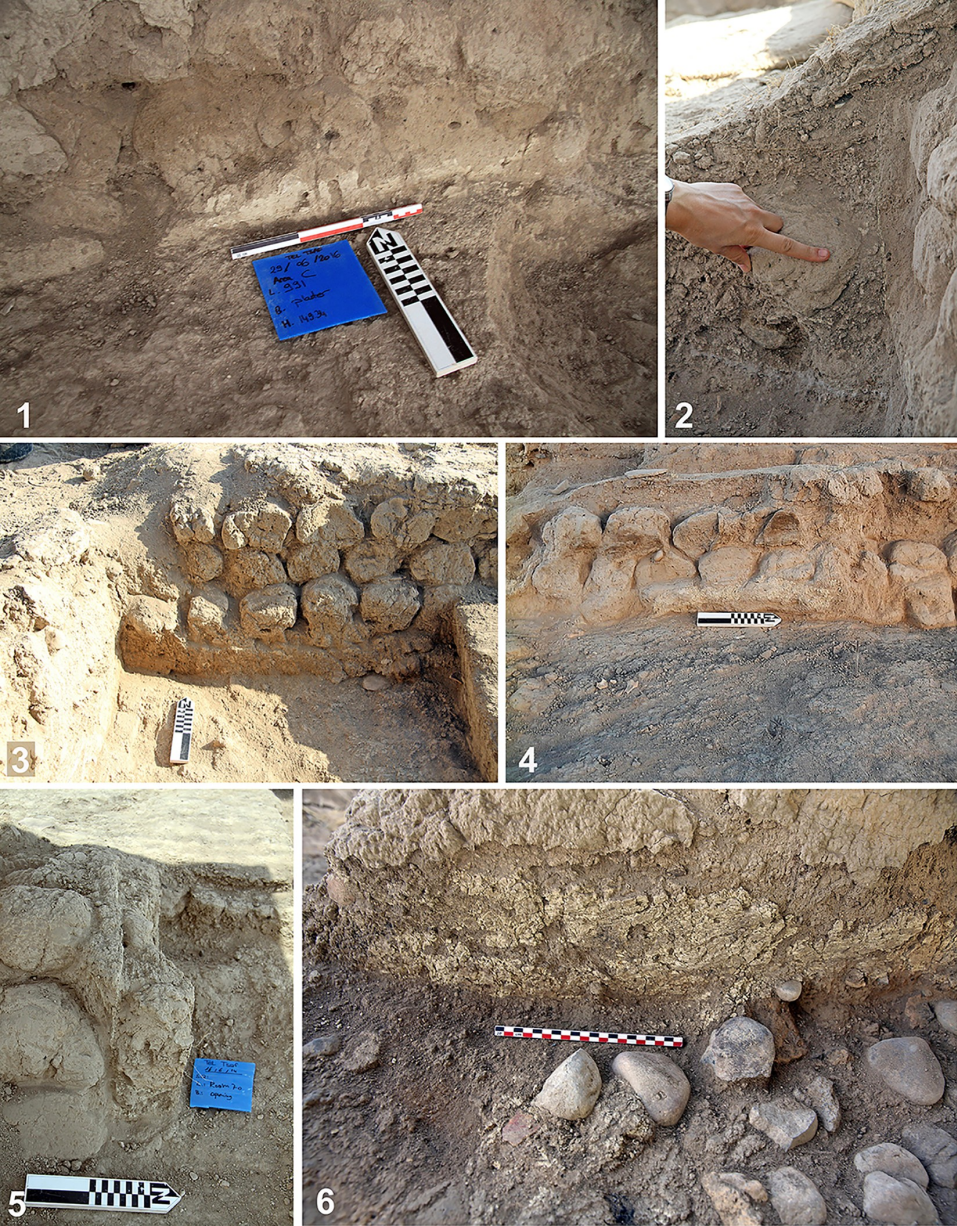
Examples of the plaster coatings of the interior walls of Room C70 and exterior wall of Structure 286. Note the variety of thicknesses.

Plastering of floors, walls (internally and externally), installations and silos was common. Plaster was used as lining and coating to maintain the integrity of the sun-dried mudbricks and prevent degradation from rain and wind. This coating was frequently formed from mud plaster and rarely lime plaster (Fig 8). The thickness of the plaster lining on the floors or walls varies, and while it generally ranges between 1–4 cm thick, the coating can be as thin as 1–2 mm (Fig 8). It is likely that sediment from the Lisan formation, characterized by marls and calcareous silt loams, was used to plaster walls and floors in order to give the structure a white appearance. As in modern times, white plaster, wash or paint may accord a special appearance to buildings or an entire village.

The mudbricks at Tel Tsaf were usually oval or loaf-shaped and handmade, with no clear evidence for the use of a mold. Several sizes were noted. The largest group of mudbricks were ca. 35–40 cm long, 15–20 cm wide and 15–20 cm thick, but smaller, usually shorter versions, were also common. Some bricks show a certain degree of exposure to heat, but this usually reflects post-production exposure to occupational or post-occupational burning activities. The bricks have a silty texture and do not vary significantly in color, ranging between 10YR 7/2 (light grey, RGB 187, 171, 150) and 10 YR 6/2 (light brown, RGB 161, 145, 125).

### The mudbricks composition

The mudbrick micromorphology fragments identified by thin sections were characterized by sharp well defined edges and densely packed fine sediment, and were clearly identifiable in 25 thin-section samples. The mudbricks include sub-rounded quartz and basaltic sands with water-laid calcareous inclusions and red chert. Mudbricks are generally characterized by plant voids, suggesting the addition of reeds, stems, grasses or seed husks as chaff. In addition, small quantities of micro-artefacts with rounded edges were added as temper to mudbricks, including flint debitage, charred wood, shell fragments, land snails, ceramic grog, and bone fragments.

**Fig 9:**
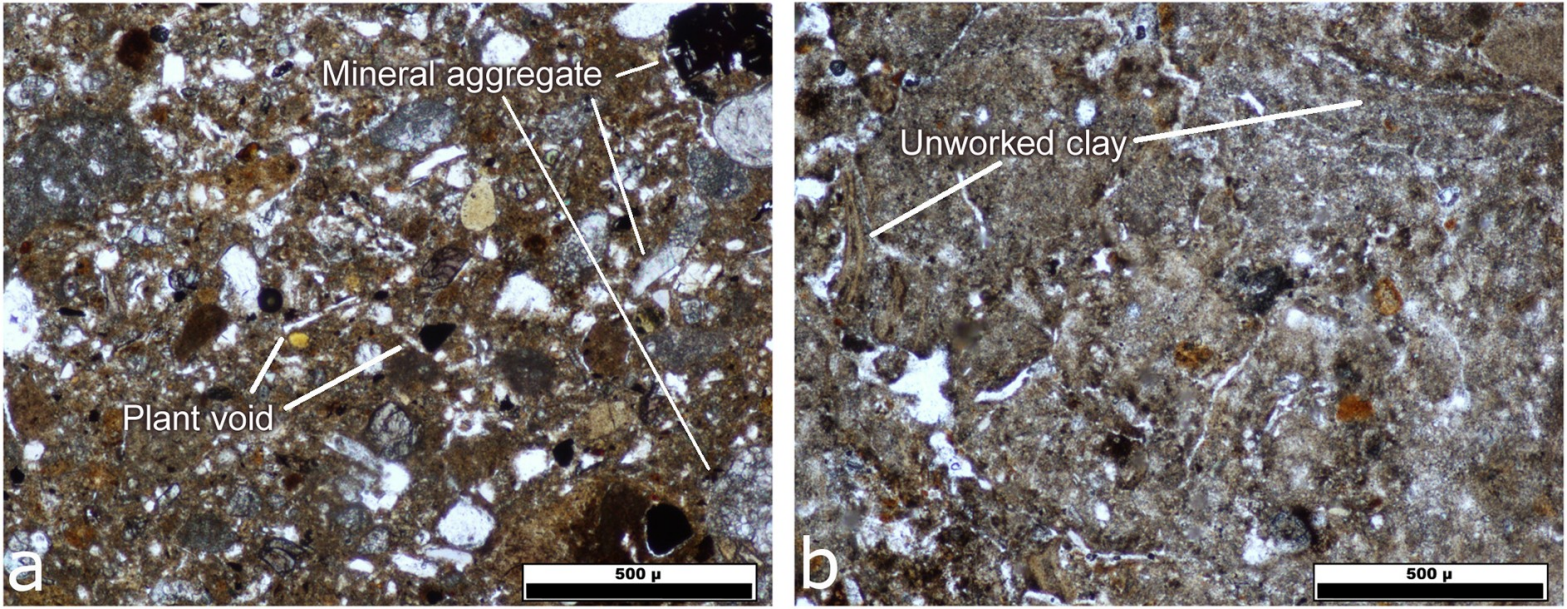
A) Thin section of a well-made mud brick. B) Thin section of a crudely-made mudbrick.

The mudbrick fragments can be classed into two broad categories: well- and crudely-made. The well-made bricks contain a densely-packed homogenous fine-fraction with some disoriented plant voids and relatively well-sorted temper inclusions (Fig 9A), and fragments were identified in all contexts and phases sampled. This technique is consistent with mudbrick production at other sites in the Near East [88–90]. The production strategy aims to lessen the chance of fissures forming during the initial drying process and, thus, enhance the stability of the mudbricks [91–92]. Crudely-made mudbricks are indicated by a less homogenous matrix with poorly sorted sediment and anthropogenic inclusions and occasional pieces of unworked clay (Fig 9B). The inclusion of unworked clay indicates that sediment was taken from an in situ context and not worked prior to its addition and that the brick was not kneaded well enough to break it up. This suggests that time and effort were main factors in quality rather than raw material choice. Brick fragments of this type were identified in three samples taken from the interior of Silo 339 in Building CI.

**Fig 10:**
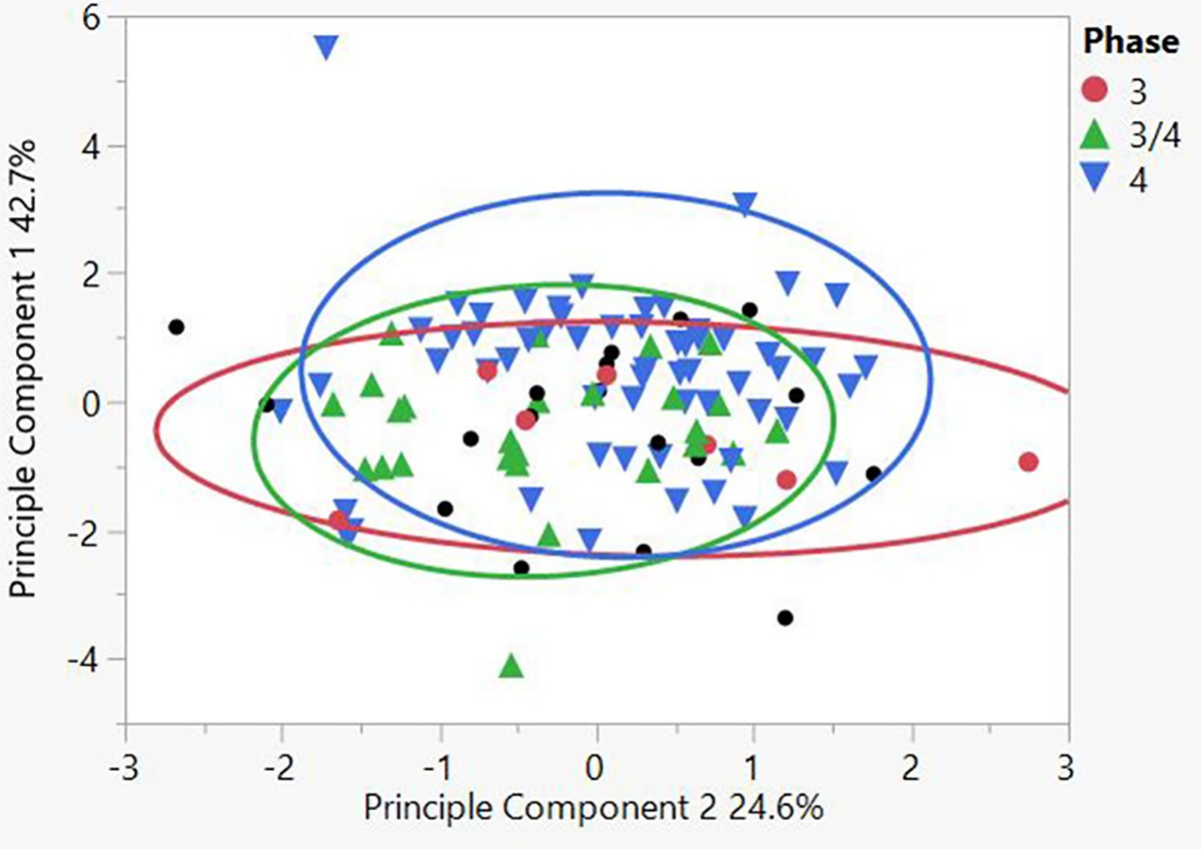
PC 1 compared to PC 2 in a 95% density ellipse, grouped by phase (N = 111).

**Fig 11:**
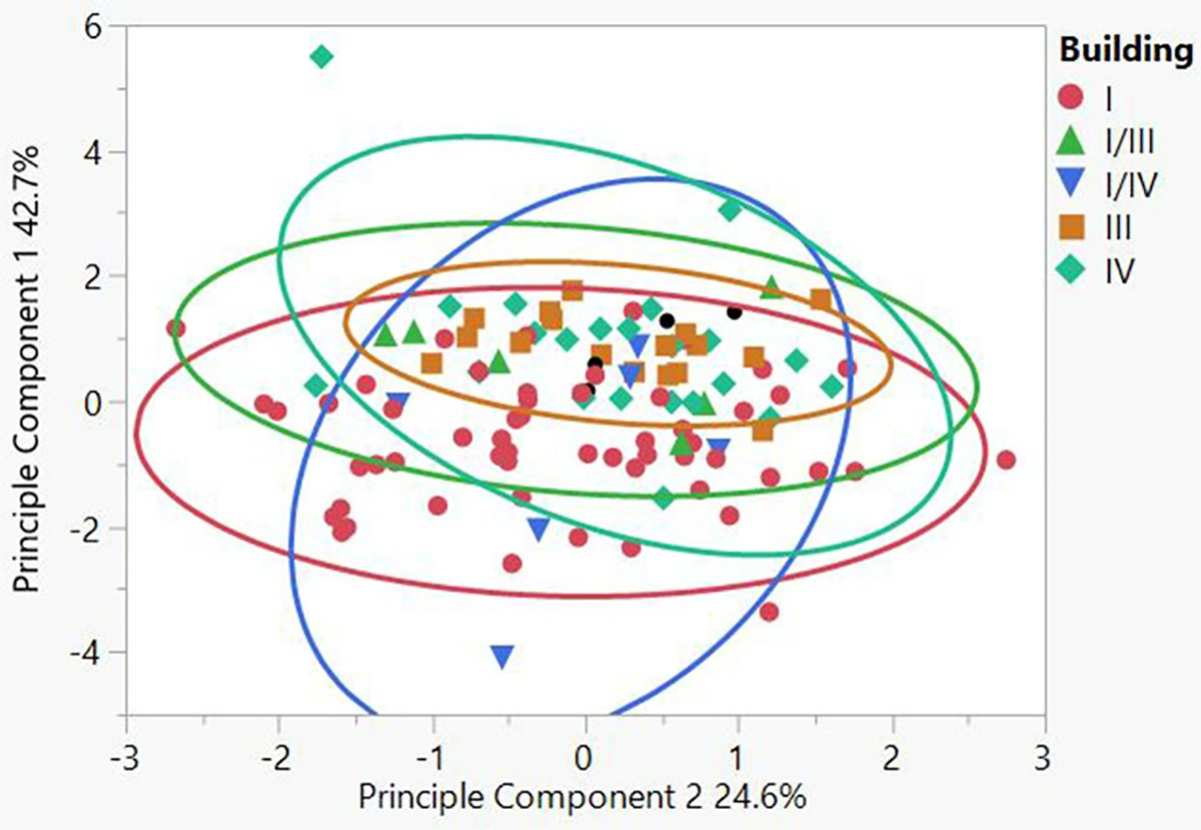
PC 1 compared to PC 2 in a 95% density ellipse, grouped by building (N = 111.).

**Fig 12:**
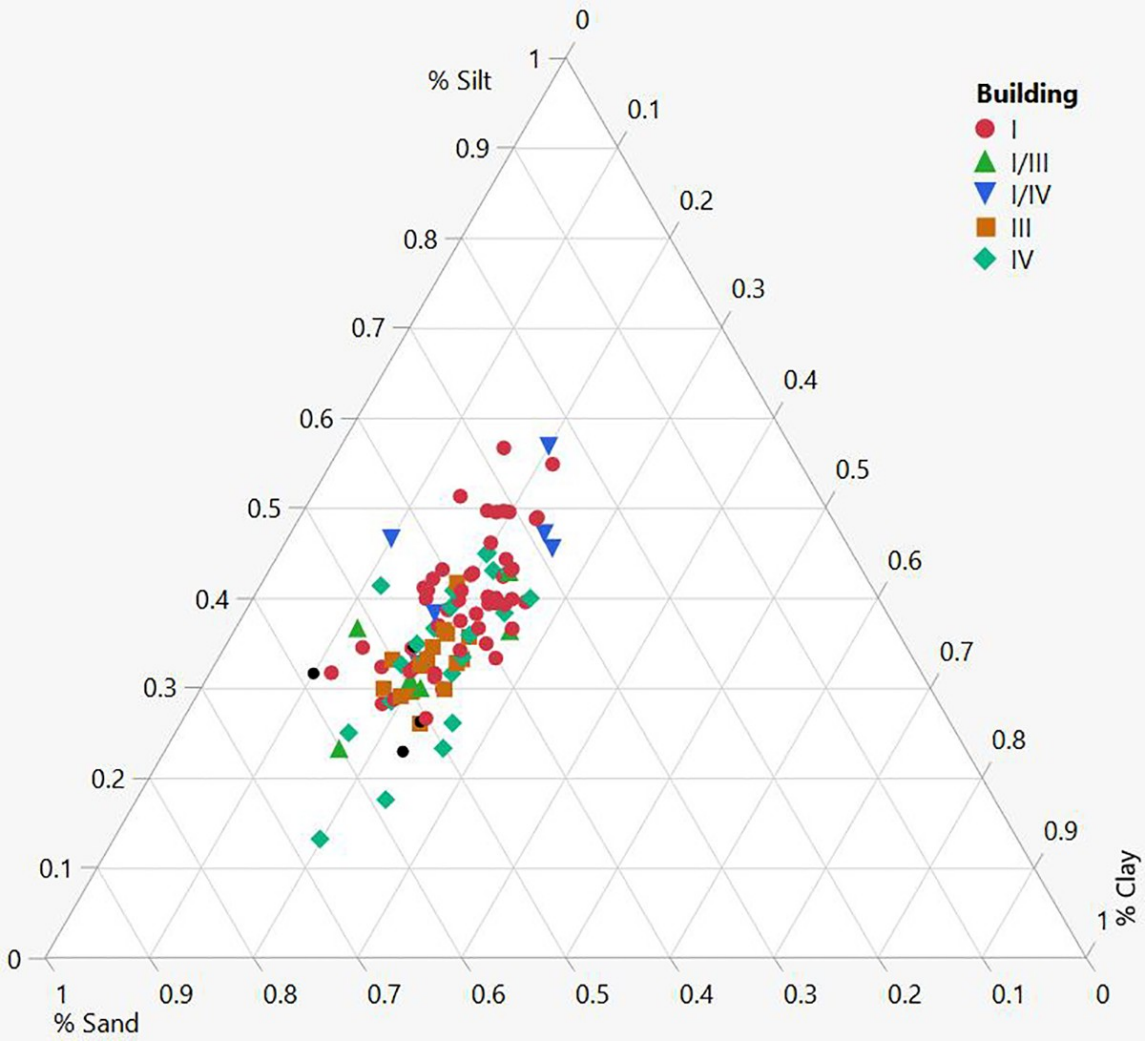
Ternary diagram showing textural variation in the mudbrick assemblage (N = 111).

From the total of 111 samples, 104 could be securely attributed to a specific building phase, and ca. 90 could be attributed to a specific building complex as not all structures are clearly assigned to a specific chrono-spatial unit. The results of the geoarchaeological analyses reveal a remarkable similarity between all buildings and diachronic compositional consistency. There is insignificant variability in three of the four variables, namely organics, carbonates, and magnetic susceptibility, but there is variation in particle distribution (Tables 3 and 4). None the less, the internal similarity of the assemblage is clear (Figs 10 and 11). Principle component 1 (PC1) is compared against component 2 (PC2) in a bivariate plot, first grouped by phase and secondly by building numbers in a 95% density ellipse. There is a strong compositional overlap between all the mudbricks, both diachronically and synchronically. A moderate distribution of brick textures throughout the assemblage was also noted. The average grain size distribution is 42% sand, 37% silt and 21% clay, but the sand fraction ranges between 23–67% (Fig 12). There was no significant size variation between site mudbrick features, and the sediments range from well to moderate to poorly sorted. Well-sorted includes mudbricks with sub-rounded sand grains, while poorly-sorted includes sand with angular and sub-angular grains.

**Table 3:** Average values of each measured variable in each phase.

Phase	Magnetic susceptibility	CaCO3 equiv.	Organic content	% Sand	% Silt	% Clay	No. Samples
**3a**	13.29	3.25	18.92	43.48	34.16	22.36	7
**3–4**	12.36	1.62	19.00	37.39	40.73	21.88	29
**4**	13.14	0.96	20.01	44.56	35.65	19.80	28
**4a**	13.27	0.75	21.76	45.28	39.79	14.93	2
**4a-4b**	14.11	2.81	19.21	42.78	38.95	18.27	22
**4b**	14.12	1.27	20.01	44.56	34.03	21.41	8
**4b-4c**	14.99	1.14	19.19	47.14	32.43	20.43	6
**4c**	13.99	0.88	20.16	38.95	38.76	22.29	8
**Min.**	12.36	0.75	18.92	37.39	32.43	14.93	
**Median**	13.64	1.20	19.61	44.02	37.20	20.92	
**Max.**	14.99	3.25	21.76	47.14	40.73	22.36	
**St.Dev.**	0.75	0.88	0.88	3.07	2.91	2.37	

**Table 4:** Average values of each measured variable represented by each building.

Bldg.	Magnetic susceptibility	CaCO3 equiv.	Organic content	% Sand	% Silt	% Clay	No. Samples
**I**	12.11	2.14	19.45	39.29	39.91	20.80	56
**I/III**	13.74	1.09	19.59	50.04	31.90	18.06	6
**I/IV**	12.15	2.43	20.29	34.80	44.15	21.05	6
**III**	14.40	1.06	19.65	47.92	32.25	19.82	17
**IV**	14.69	1.00	20.03	44.81	34.09	21.11	21
**Min.**	12.11	1.00	19.45	34.80	31.90	18.06	
**Median**	13.74	1.09	19.65	44.81	34.09	20.80	
**Max.**	14.69	2.43	20.29	50.04	44.15	21.11	
**St. Dev.**	1.09	0.61	0.31	5.61	4.80	1.15	

The bulk sediment samples, processed through mechanical sieves, produced grain-size distributions similar to those in the mudbricks. There are some fluctuations in proportion of silt/clay (<64μm), sand (>250μm and <1mm) and micro-artefacts (>1mm). In all contexts the sediments suggest local production and exploitation of a similar source material. The mudbricks contain a slightly higher than average proportion of sand fractions, which can likely be attributed to choices related to tempering. However, the overall similarity of grain sizes and distribution provides no evidence for non-local building materials. A qualitative analysis of the sand fractions (>250 μm) and micro-artefacts (>1 mm) reveals a similar result.

## Discussion

Mudbrick architecture appears in tandem with early farming communities in the Jordan Valley, suggesting that the potential and advantages of using mudbricks for construction were recognized during the early Neolithic (e.g., PPNA Jericho). While a considerable amount of stone building techniques existed from beginning of the PPNA, mudbrick architecture likely developed due to the lack of suitable stones for use as building material [6]. Such a situation is given in parts of the Jordan Valley (specifically in areas closer to the Jordan River); thus, the region was likely a center of experimentation and improvement of mudbrick technology. Ancient technology however is not developed sequentially, and the reasons behind improvement innovations seem often esoteric from a modern perspective [93]. Within the Jordan River area, mudbricks continued to form a major component in construction throughout most of the later prehistoric and historic periods (e.g., Tel Rehov).

Tel Tsaf is a relatively late site in the development of mudbrick technology, and as we have shown, a very homogeneous and standardized production and implementation of mudbricks was already established by the late 6^th^ and early 5^th^ millennia cal BCE. These technologies were deeply integrated into the construction of rooms and walls, but also platforms, installations and silos. The utilization of mudbricks for the latter as well as the standardization of brick recipes are however novelties defined by this period. The homogeneous production technique was demonstrated at the site over several hundred years.

Tel Tsaf mudbricks were sun-dried, suggesting that there was no need for burnt/baked mudbricks at the site. This saved energy required for acquiring fuel for the burning but also necessitated regular maintenance, including plastering of the mudbricks to prevent decay during the rainy season. This maintenance demanded its own investment of energy and time. The lack of a stone foundations intended to impede water seepage that would undermine the mudbrick superstructure and provide stability to the structure is also of note. Instead, the wall thickness was selected to form a firm structure in addition to aid in supporting the superstructure (i.e., increasing their load-bearing capacity).

One of the most interesting outcomes of the current study is that despite variations in architectural features (in terms of size, contour and construction method), a single recipe was utilized for the production of most mudbricks at the site. 95% of the 111 studied mudbrick samples share a similar composition regardless of provenance, context or temporal phase. The remaining 5% of mudbrick samples differ solely in their sand fractions. The mudbrick compositions also confirm that the building materials were likely made from locally-sourced materials of the Lisan formation with alluvium, aeolian and fluvial deposits composed largely of calcite, limestone and sandstone.

Three of the four variables used to identify mudbrick compositions have statistically insignificant variation (magnetic susceptibility, organic content and CaCO_3_ values). Magnetic susceptibility and CaCO_3_ values are both indicators of source material, whereas organic content, particle size distribution and micro-artefacts can be manipulated by a brick-maker as tempering materials. Thus, considering that most of the architecture so far uncovered in the site’s upper levels dated between ca. 5200–4700 cal BCE [59–60], the results show that the mudbrick composition at Tel Tsaf was stable for at least 500 years.

The absence of independent, time specific or spatially exclusive recipes and the consistency of mudbrick-production technology during all phases of occupation at Tel Tsaf implies shared production and technological knowledge but also relates to the availability of excellent raw material that required minimal modification. Further consistency in mudbrick composition may also be contributed to the recycling of old mudbricks or mudbrick debris. Ethnographic observations in Pakistan [94] illustrate how once the upper courses of mudbrick walls collapsed, these were soaked in water overnight, forming good quality material for the production of new mudbricks. This process would create similarities between buildings if the mudbricks of one structure were recycled into the walls of another. While the sediments would be reused, minor variation would form by the types of organic temper used. However, the apparent lack of change to the *chaîne opératoire* over several centuries demands further explanation.

Evidence does not support that brick production was specialized or that access to local resources was restricted. Considering this, one possible explanation for such long-term continuity would be that mudbrick production and construction was a communal task where an ideal mudbrick recipe was shared by the whole community and perpetuated by transmission its younger members. Alternatively, the archaeological context of large-scale storage implies a certain form of control that might also encompass labor. In other words, bricks were not made by every household individually. This scenario would also involve the mobilization of larger groups for construction work, but the uniformity of mudbrick recipes was then the result of centralized control.

The types of materials used to form and construct architecture are not only reflections of available resources but also of specific and intentional cultural choices (see [95]). While it is possible that the people settled at Tel Tsaf had little choice but to use locally available raw material for construction and that they may have recycled old building materials, clearly they were able to fully harness these readily available sediments. Furthermore, it seems that the need to protect the mudbrick walls forced the settlers at Tel Tsaf to develop maintenance technologies implemented on a regular basis, probably before the winter rains appeared. The rich architectural record of Tel Tsaf thus suggests that, while sundried mudbrick constructions were favored by the local conditions, it probably entailed high investment in maintenance. This challenges the apparent common-sense conclusion that mudbrick construction was ideally suited for the settlement. The long tradition of mudbrick buildings at Tel Tsaf suggests that there must have been advantages in this technique that made it worthwhile to continue production within very strict parameters. No matter how the organization of the work is modelled, the data highlights that the necessity of constant care for structures and installations was preferred to the use of other construction materials such as wooden logs and stones that were unavailable at the site vicinity. Thus, while the creation of buildings from the locally available clay might be seen as a simple common-sense solution, it traded short-term costs for long-term costs.
